# Effect of Crumble-Pellet and Mash Diets with Different Levels of Dietary Protein and Energy on the Performance of Broilers at the End of the Third Week

**DOI:** 10.4061/2010/328123

**Published:** 2011-01-23

**Authors:** S. Jafarnejad, M. Farkhoy, M. Sadegh, A. R. Bahonar

**Affiliations:** ^1^Faculty of Veterinary Medicine, University of Tehran, P.O. Box 14155-6453, Tehran, Iran; ^2^Department of Animal and Poultry, Health and Nutrition, Faculty of Veterinary Medicine, University of Tehran, P.O. Box 14155-6453, Tehran, Iran; ^3^Department of Food Hygiene, Faculty of Veterinary Medicine, University of Tehran, P.O. Box 14155-6453, Tehran, Iran

## Abstract

This experiment was conducted to investigate the effect of the form of diets with different levels of protein and energy on broilers performance at the end of the third week. A total of 2800 male broiler chicks were fed with two forms of diet (mash and crumble-pellet), two levels of protein (23% and 21% CP), and two levels of energy (3200 and 3000 Kcal/Kg ME) from 1 to 21 days of age. The bodyweight (BW) and Feed conversion rate (FCR) were affected by the form of diet with the crumble-pellet form being better (*P* < .001). The diet with high protein significantly increased BW and decreased FCR (*P* < .001). The different levels of energy did not affect FCR and BW in crumble-pellet diet but should a significant effect on them in mash diet (*P* < .05). There were no significant interactions for any of the parameters tested except for interactions between energy and feed form. BW and FCR were improved by energy when diets were fed in the mash form (unlike the crumble-pellet form) at all ages. It is concluded that feeding crumble-pellets from 1 to 21 days of age improved BW and FCR and that an increase in the protein (unlike energy) content of the diet increased the performance of the chickens at the end of the third week.

## 1. Introduction

Successful broiler development is dependent on optimal feed intake throughout the growing period. Optimal feed intake is dependent on a number of factors such as environmental temperature, and diet nutrient density, and physical feed quality is considered to have a very significant impact on broiler growth. Energy and protein are very important nutrients for broilers like other living creatures. Energy is required for body functioning and protein is an essential constituent of all tissues of animal body. Protein having major effect on growth performance of the bird is the most expensive nutrient in broiler diets [1]. It is a widely accepted principle in poultry nutrition that dietary energy and the essential nutrients must be considered as an entity. To ensure maximum utilization of energy, protein, and every nutrient of the diet, a right proportion of these nutrients are necessary for optimum growth of the birds and for minimization of the surplus use of vital dietary component, and because the first few days after hatch now represent a greater percentage of a broiler's lifespan than any time in history, it is critical that the bird be given every opportunity to get off to a good start.

The physical form of feed (mash and pellet) is a crucial factor in meat yield of broiler. Mash is a form of a complete feed that is finely ground and mixed so that birds cannot easily separate out ingredients; each mouthful provides a well-balanced diet. Mash diet gives greater unification of growth and less death loss and is more economical. However, ground feed is not so palatable and does not retain its nutritive value so well as ungrounded feed [2]. Pellet system of feeding is really a modification of the mash system. It consists of mechanically pressing the mash into hard dry pellets or “artificial grains”. It is generally accepted that, compared to mash, the feeding of pellets improves broiler growth rate with an increased feed intake [3–6]. Reasons for the enhanced performance may be due to increased digestibility, decreased ingredient segregation, reduction of energy during prehension, and increased palatability [7], but feeding pelleted rations is not enough to ensure enhanced performance of poultry. The quality of pellets must be taken into account also [8]. 

Crumble also is a type of feed prepared at the mill by pelleting of the mixed ingredients and then crushing the pellet to a consistency coarser than mash. Recently this form of feed has become popular in broiler production due to its convenience of feeding. Choi et al. [4] reported that chicks fed the crumbled starter diet consumed more feed. 

The aim of this study was to investigate the effects of different levels of energy, protein and different feed forms (mash and crumble-pellet), and interactions between them on the performance of broilers in starter period upon the broilers performance.

## 2. Materials and Methods

A total of 2800 commercial male broilers (Ross 308) were used in a completely randomized design with eight treatments and seven replications. One-day-old broilers (average initial bodyweight of 45 g) were allocated to dietary treatments. There were 50 birds per replicate, and the stocking density was 17 birds/m^2^. Room temperature was maintained at 33°C during the first week, and it was decreased 3°C each week till it reached 27°C at the end of the third week. All diets were corn-soybean based. Feed and fresh water were supplied ad libitum during the experiment. A 24-hour light was made available to the chicks throughout the experiment. The experiment was used in a factorial arrangement 2 × 2 × 2 with two forms of diet (mash, crumble and pellet) (crumble and pellet form of diet used, respectively, in the first 10 days and 10–21 days old), two levels of protein (23% and 21% CP), and two levels of energy (3200 and 3000 Kcal/Kg ME). The ingredient composition and analysis of the basal diets are shown in Table 1. Feed intake and weight gain were recorded at the end of first, second, and third week, and feed conversion ratio (FCR) was calculated. The experimental design was analyzed statistically using ANOVA technique. The GLM is used to summarize data in some parameters that are pertinent to the experiment.

## 3. Results and Discussion

Data on weight gain and FCR at the end of first, second, and third week are presented in Table 2. The weight gain was significantly greater in broilers fed crumble-pellet diets than mash diets when assessed over trial period (*P* < .001). T1 (crumble-pellet form with 3200 Kcal/Kg and 23% protein) had the highest weight gain (951 g) and the best FCR (1.31). The results were in accordance with those of van Biljon [9] who reported that chickens on the crumble-pellet dietary regimen were significantly heavier at 42d when compared with birds fed either all-mash or ground crumble-pellet regimen. Jahan et al. [2] reported that the highest body weight gain was observed in the crumble group throughout the experiment period, but these data were statistically similar with pellet group from 5 to 8 weeks of age. Allerd et al. [10] also reported that chicks grew faster when fed as pellets or crumbles than when the same diets were fed as mash. In accordance with other authors results [3–6, 11] the feeding of pellets, compared to mash, improved broiler growth rate, which was associated with an increased feed intake and improved feed conversion efficiency. Also, Agah and Norollahi [12] reported that the usage of mash diet in the first ten days and pellet diet in other growing periods could be having the best FCR and the highest weight gain which is in agreement with the result of this study. 

Fairfield [13] noticed that pelleting of feed also provides the benefits of increasing the bulk density of feed, improving feed flow ability and providing opportunities to reduce feed formula costs through the use of alternative feed ingredients.

Maximum weight gain was observed in birds fed on diet containing 23% CP on both crumble-pellet and mash diets in all weeks (*P* < .001). Birds fed dietary energy levels of 3200 Kcal ME/kg gained significantly more weight than birds fed 3000 Kcal ME/kg in the mash diets at second (*P* < .05) and third (*P* < .001) weeks but no difference in BW was noted for birds fed the pellet diets with the different levels of metabolizable energy in all weeks.

 Metabolizable energy had a significant interaction with feed form for all of the performance parameters studied. There were interactions between metabolizable energy and feed form on BW and FCR at all ages examined. Both main effect and interactive data are shown in Table 3. Body weight was not affected by metabolizable energy when diets were fed in pellet-crumble form. In contrast, BW decreased and also FCR increased with reduced energy when diets were in mash form (Table 3).

Conflicting results on the interaction between feed forms and energy have been reported. Whereas some trials have showed significant interactions [14, 15], others did not [16]. Scott [17] has reported a decrease in growth rate with decreasing nutrient density and metabolizable energy when diets were fed in mash form which is similar to the findings of this research. This relationship was likely a reflection of the reduced ability of birds to eat the bulkier, and possibly less palatable, low-density mash ration. The interaction between nutrient density and feed form on feed intake supports this conclusion.

 It was also observed that birds fed on low ME and high CP diets gained more weights as compared to those fed high ME and low CP diets. These findings are contrary to those reported by Holsheimer and Veerkamp [18], claiming that the normal-CP diets gave significantly higher gains than the high-CP diets at 6, 7, and 8 weeks of age. The results were also in accordance with those of Onwudike [19] who reported an increased daily weight gain with 2800 Kcal ME/kg and dietary protein of 22% (in compare to 20%) in broiler chickens. The findings of this study were in close agreement with those of Leeson et al. [20] who found that high-energy and low-protein diets (3000 Kcal ME/kg and 20% CP) depressed the growth of broilers. They also reported significantly higher body weight gains with 24 and 22% CP compared to 20%. However, Leeson et al. [21] reported that body weight gain and growth rate were unaffected by the level of the energy in diet of broiler chicks that is in agreement with the result of this study. Han et al. [22] observed no difference in body weight gain in low-protein diets for broilers when being fortified the low-CP diets with essential amino acids. So, it seems that growth depression due to low-protein diets may be due to low-amino-acid profile of such diets. In this trial, FCR was affected by the form of diet with the pellet form being better in all weeks (*P* < .001).

On the crumble-pellet and mash diets, it was noted that birds fed on diets containing 23% CP had lower FCR than the birds fed on diet containing 21% CP, specially at the 2nd and 3rd week (*P* < .001). A similar response to dietary energy was observed for FCR in the mash diets at the 1st week (*P* < .01) and at the 2nd and 3rd weeks (*P* < .001), but the effect of different levels of energy on FCR in the pellet diets was insignificant except for the 2nd week (Tables 2 and 3).

The results of this study are in agreement with those of Onwudike [19] and Temim et al. [23] who reported that feed efficiency was improved with increasing dietary CP levels for broiler chicks. The results were also in accordance with those of Jackson et al. [24] who noted that feed efficiency increased with increasing levels of dietary protein or energy. The findings of this study are supported by Leeson et al. [20] who reported improved feed efficiency with 22 and 24% CP and 3000 Kcal ME/kg as compared with diets with 20% CP and 2600 Kcal ME/kg. Ferguson et al. [25] reported an increase in FCR when CP was reduced from 21.5 to 19.6% in a six-week broiler growth trial. Similarly, Si et al. [26] reported a significant effect of dietary CP on FCR. Nawaz et al. [27] also reported that low-ME and high-CP diets promised optimum performance for broiler chicks at both starter and finisher phases which is in agreement with the result of this study. 

It is concluded that feeding crumble-pellets from 1 to 21 days of age improved BW and FCR and that an increase in the protein (unlike energy) content of the diet increased pellet performance at the end of the third week. In conclusion, it is obvious that feeding crumble-pellets from 1 to 21 days of age improves BW and FCR and that an increase in the protein (unlike energy) content of the diet increases chickens performance at the end of the third week.

## Figures and Tables

**Table 1:** Ingredient composition and nutrient content of experiment diet (as fed basis).

	Crumble-pellet diets				Mash diets			
Ingredients (g/100 gr)	1	2	3	4	5	6	7	8

Yellow corn	48.4	51	54.8	57.2	48.4	51	54.8	57.2
Soybean meal	37.9	40.5	32.4	35.2	37.9	40.5	32.4	35.2
Oil	7.15	4	6.1	3	7.15	4	6.1	3
Monocalcium phosphate	1.45	1.4	1.5	1.45	1.45	1.4	1.5	1.45
Limestone	1.7	1.7	1.7	1.7	1.7	1.7	1.7	1.7
Salt	0.4	0.4	0.4	0.4	0.4	0.4	0.4	0.4
Dl-methionine	0.3	0.3	0.3	0.3	0.3	0.3	0.3	0.3
Lysine	0.21	0.15	0.27	0.23	0.21	0.15	0.27	0.23
Vit. & min. premix^1^	0.5	0.5	0.5	0.5	0.5	0.5	0.5	0.5
Wheat gluten	2	0	2	0	2	0	2	0

*Calculated composition*								
Crude protein (%)	23	23	21	21	23	23	21	21
ME (kcal/kg)	3200	3000	3200	3000	3200	3000	3200	3000
Calcium (%)	1	1	1	1	1	1	1	1
Av. phosphorus (%)	0.45	0.45	0.45	0.45	0.45	0.45	0.45	0.45
Sodium (%)	0.17	0.17	0.17	0.17	0.17	0.17	0.17	0.17
Methionine (%)	0.64	0.64	0.62	0.62	0.64	0.64	0.62	0.62
L-Lysine (%)	1.35	1.35	1.25	1.25	1.35	1.35	1.25	1.25
Methionine + cystine (%)	1	1	0.95	0.95	1	1	0.95	0.95

^1^Each kg of premix provided vitamin A, 10000 IU; vitamin D**_3_**, 2500 IU; vitamin K, 2.4 mg; vitamin E, 44 IU; biotin, 0.1 mg; folic acid, 2.0 mg; niacin, 25 mg; calcium pentothenate, 14.32 mg; pyridoxine, 3.10 mg; riboflavin, 5 mg; thiamin, 1.2 mg; vitamin B**_12_**, 10.5 *μ*g; Fe, 85 mg; Mn, 125 mg; Cu, 7.8 mg; Se, 0.09 mg; Zn, 60 mg; choline chloride, 5.5 mg.

**Table 2:** Weight gain and feed conversion ratio (FCR) of broiler chickens fed on mash and crumble-pellet as affected by various metabolizable energy (ME) and crude protein (CP) levels of the diets* at the end of the third week (mean + SE).

Treatments	Protein (%)	Energy (Kcal/Kg)	Diet form	Weight gain (g)			FCR (g/g)		
				1st wk	2nd wk	3rd wk	1st wk	2nd wk	3rd wk

1	23	3200	A	177^ab^ ± 1.7	486^a^ ± 4.8	951^a^ ± 5.3	.84^c^ ± 0.004	1.13^d^ ± 0.009	1.31^e^ ± 0.013
2	21	3200	A	172^b^ ± 2.1	464^b^ ± 4.8	905^b^ ± 6.2	.86^c^ ± 0.003	1.15^d^ ± 0.009	1.35^d^ ± 0.009
3	23	3000	A	179^a^ ± 3.1	488^a^ ± 6.8	936^a^ ± 10.5	.85^c^ ± 0.005	1.14^d^ ± 0.011	1.30^e^ ± 0.012
4	21	3000	A	177^ab^ ± 2.2	475^ab^ ± 4.2	913^b^ ± 6.4	.88^c^ ± 0.018	1.18^c^ ± 0.004	1.37^cd^ ± 0.007
5	23	3200	B	162^c^ ± 1.8	438^c^ ± 4.6	842^c^ ± 4.4	.87^c^ ± 0.024	1.18^c^ ± 0.016	1.36^d^ ± 0.014
6	21	3200	B	156^de^ ± 1.6	422^d^ ± 3.1	818^d^ ± 8.5	.89^bc^ ± 0.020	1.21^c^ ± 0.011	1.39^c^ ± 0.004
7	23	3000	B	158^cd^ ± 1.8	420^d^ ± 3.3	798^d^ ± 6.4	.94^ab^ ± 0.032	1.24^b^ ± 0.013	1.43^b^ ± 0.013
8	21	3000	B	151^e^ ± 1.6	401^e^ ± 2.4	757^e^ ± 8.3	.95^a^ ± 0.007	1.28^a^ ± 0.005	1.49^a^ ± 0.008

*P* value				.000	.000	.000	.000	.000	.000

^a-b-c-d^Means with lacking common superscripts differ significantly.

*Mean of 7 replicates having 50 birds in each replicate.

**A; crumble-pellet diet, B; mash diet.

**Table 3:** The effect of energy, protein, and feed form and their interactions on the performance of broilers fed mash and crumble-pellet diet treatments.

	E∗P∗F	P∗F	E∗F	E∗P	F	P	E
BW(1st wk)	NS	NS	**	NS	***	***	NS
BW(2nd wk)	NS	NS	***	NS	***	***	*
BW(3rd wk)	NS	NS	***	NS	***	***	***
FCR(1st wk)	NS	NS	*	NS	***	NS	**
FCR(2nd wk)	NS	NS	**	NS	***	***	***
FCR(3rd wk)	NS	NS	***	NS	***	***	***

NS = nonsignificant; *P < .05; **P < .01; ***P < .001.

E: energy; P: protein; F: feed form; E∗P: counteraction between energy and protein; E∗F: interaction between energy and feed form; P∗F: interaction between protein and feed form; E∗P∗F: interaction between energy, protein, and feed form.
